# Farmers’ Participatory Plant Selection of Lablab (*Lablab purpureus* (L.) Sweet) in Tanzania

**DOI:** 10.3389/fpls.2022.784032

**Published:** 2022-06-23

**Authors:** Fanuel K. Letting, Pavithravani B. Venkataramana, Patrick A. Ndakidemi

**Affiliations:** ^1^Department of Sustainable Agriculture, Biodiversity and Ecosystems Management, School of Life Sciences and Bio-Engineering, The Nelson Mandela African Institution of Science and Technology, Arusha, Tanzania; ^2^Department of Seed, Crop and Horticultural Sciences, School of Agriculture and Biotechnology, University of Eldoret, Eldoret, Kenya

**Keywords:** lablab (*Lablab purpureus*), participatory breeding, production practices, production constraints, trait preferences

## Abstract

Farmer-participatory breeding approach is an important component in the crop improvement of lablab (*Lablab purpureus* (L.) Sweet). The study was carried out to obtain the knowledge, practices and preferences of lablab through 31 lablab growing-farmers from Arusha, Kondoa, Karatu, Same and Babati districts of Tanzania toward initiating a lablab breeding program. Semi-structured questionnaires were administered and focused group discussions were held to collect data on the socio-demographic factors, production practices, constraints and farmer’s preferred traits of lablab. Selection of preferred traits and accessions was also done by the farmers in the field. Results showed that the chief constraints of lablab production are pests and diseases, poor marketability, low seed quality, inadequate rainfall, expensive agrochemicals, low yield, and poor storage facilities. The major pests are pod borer (field) and bruchids (storage). Preferred traits for lablab improvement include the development of insect pests and disease-resistant varieties, early maturing, high yield, black colored seed for market, short cooking time, and dense foliage. Genotypes EK2, D360, HA4, and D96 with preferred traits were identified by farmers, which forms critical decisions in crop improvement. This study describes the current view of lablab production and generates the understanding of farmers’ perceptions and preferences vital for breeding priorities and programs to increase its production, utilization and consumption.

## Introduction

Advanced plant breeding incorporates genomics and phenotyping which have fast-tracked breeding through the application of molecular marker technology and genome-wide selection methods which have been found to increase the genetic gains in some crops (Watson et al., 2018). These breeding techniques are regarded as a centralized approach where knowledge and management of the genetic resource remains solely with the breeder/researcher without the decisions of the end-users (Fadda et al., 2020). Despite the immense contribution of these modern breeding approaches, the adoption, utilization, and adaptation to adverse climatic conditions of these elite genetic resources remain a challenge (Sanghera et al., 2013). The lopsided problem-solving method has neglected the importance of the end-user, the farmer, culminating the adoption of improved varieties by farmers in the production plan. A strong collaboration between researchers and farmers is required.

The participatory plant breeding approach involves collaboration between researchers and farmers in the genetic improvement of crop species by enabling diffusion, adoption, and incorporation of the improved crop varieties at the farmers’ level. The farmer and the researcher bring different perspectives on improving the crop through knowledge, skills, and experience (Hoffmann et al., 2007; Weltzien and Christinck, 2017), and they contribute during fundamental decision-making phases. Farmer participation plays a crucial part at all stages of the breeding program, from the description of objectives, experimental designs, selection of experimental sites, choice of germplasm resources, field trials, seed production and transfer, and adoption at the community level (Singh et al., 2021). The participation of farmers can be classified into functional and empowering participation (Bhargava and Srivastava, 2019). In functional participation, breeders tailor their approach based on the farmers’ economic resources while ensuring the breeding objectives are achieved. In empowering participation, the farmers are equipped with skills and knowledge vital for breeding to allow active participation in the breeding program (Bhargava and Srivastava, 2019). This participation plays a significant role in the utilization of the released varieties.

Farmer knowledge and skills have contributed to the modern breeding techniques which were accelerated by the advent of the green revolution. The green revolution transformed the agricultural sphere leading to the development of high-yielding, uniform, and site-specific varieties globally. Positively, it reduced the poverty levels, increased food sufficiency, and lowered food prices (Pingali, 2012). Nevertheless, the green revolution remained neutral on the concept of the conservation of biodiversity. The nexus between improving farmers’ landraces and incorporating both indigenous and scientific knowledge is pivotal in breeding programs. This allows the farmer to influence the variety adoption at the local level. Neglected and underutilized species are critical to agricultural diversity and have a rich indigenous knowledge base (Mabhaudhi et al., 2019). Among several crops considered as underutilized crops, lablab is reported as a promising species for increased agricultural systems and food security (Maass et al., 2010).

Lablab is an orphan, underutilized and neglected crop that is native to Eastern Africa (Maass et al., 2010; Letting et al., 2021). Despite its nutritional value, food (Minde et al., 2020), and economic importance, the production, and utilization of this crop are not documented. Farmers are regarded as the custodians of the important genetic resources/germplasm and crucial indigenous knowledge that are always passed from one generation to another. In Tanzania, lablab was previously grown in the northern Arumeru district in the 1930s, but declining land size, lack of research, use of low-quality landraces, and the development of high-yielding major crops have contributed to the disappearance of lablab from the biodiversity as well as the production plans by the farmers (Ngailo et al., 2003; Upadhyaya et al., 2011). According to Witcombe et al. (1996), farmer participatory breeding and participatory variety selection are fundamental when undertaking the breeding of neglected crop species and farmers’ involvement can influence the adoption rates and subsequent production of the crop.

Currently, little information on lablab production in Tanzania is known with fragmented information on its use as conservation agriculture (Shetto and Owenya, 2007; Mariki and Miller, 2017). This pertinent information on lablab production requires concerted efforts from the researchers, breeders, and farmers through a participatory approach to understand the existing knowledge at the local level and merge it with the scientific concepts of research to increase its production. Collaborations with lablab-growing farmers can allow researchers and scientists to tailor their research to the needs of farmers and set up production while increasing the chances of transfer of technologies and adoption of improved varieties. The present study, therefore, delves into assessing the production constraints, farmer preference, existing seed systems, marketing and cropping systems of lablab in selected districts in Tanzania toward developing a lablab breeding program.

## Materials and Methods

The lablab experimental field for farmers’ participatory selection was set up at the Nelson Mandela African Institution of Science and Technology (NM-AIST), Arusha (Northern part) Tanzania during the long rainy season (May–August 2020). NM-AIST lies at Latitude 03^°^02′17.0′′ S and Longitude 037^°^35′24.9′′E at an elevation of 1,106 m.a.s.l. The mean maximum temperature ranges from 22^°^C to 28^°^C while the mean minimum temperature ranges from 12^°^C to 15^°^C, respectively. Three hundred and twenty lablab accessions were planted in an augmented block design generated by the statistical tool on the website^1^ with three checks and ten blocks. The checks were replicated twice in each block and the total number of experiment units was 390. This was done on a 40 × 30 m land size divided into 10 blocks of 39 rows, each representing one accession (treatment), and 10 seeds planted on each row (one seed per hole) with a spacing of 45 cm between plants, and 70 cm between the rows and 100 cm between the blocks. Normal agronomic and crop protection practices were followed with irrigation done twice in the absence of rainfall during the vegetative and podding stages of plant growth.

### Sampling Method

Farmers for the participatory selection were purposively sampled using a multi-stage sampling technique. Thirty-one farmers from five lablab-growing regions namely, Arusha (12), Kondoa (5), Karatu (5), Same (5), and Babati (4) were invited to take part in this study at NM-AIST during the podding stage of the lablab plants in the experimental field.

Individual interviews with farmers were done with the aid of semi-structured questionnaires and focused group discussions were held to understand the farmers’ knowledge, perceptions, production constraints, preferences, and experience in lablab production before the field visit. The questionnaire consisted of socio-demographic factors, field management, processing, marketing, and storage related questions of lablab. The information on the attributes considered by the farmers for lablab varietal adoption was obtained through the discussions and the semi structured questionnaires. These attributes were used for selecting the most preferred accessions during the field visit by the farmers. Farmers were then divided into six groups each of 5 people and a trained assistant was assigned to assist during the field visit. Each group had one representative from each district as well as gender equality was considered. At first, farmers were allowed to walk in the field, looking into the performance of the individual accessions. After observing the whole field, the farmers were advised to identify the best preferred accessions (at least 5) for 11 attributes mentioned by the farmers namely, early maturity, disease tolerance, high yielding, animal feed, intercrop, food, market, pest resistance, seed color, seed shape, and soil conservation. The first five accessions with the highest number of frequencies in each trait were selected.

### Data Analysis

The quantitative and qualitative data collected from the questionnaire and focus-group discussions were coded, organized, and analysis was done using the statistical package IBM SPSS 21. The analysis included cross-tabulations and descriptive statistics computed from the data obtained.

## Results

### Demographic Description of the Respondents

Thirty-one lablab growing farmers were interviewed. A majority of them (71%) were male while (29%) were female. Male farmers outnumbered female farmers in each of the five study regions. Over 83% of the participants were more than 36 years of age. Regarding education, 74% of the respondents had primary level education, 19.4% secondary level and 3.2% had tertiary education. The main occupation of the respondents was farming, with over 90% being involved entirely in farming while the remaining 10% were also involved in other private sectors as secondary occupation. Most of the respondents were married (80%), whereas 12.9% were single and 6.5% were widowed. Seventy percent of the respondents had less than 10 years of experience in crop farming. 87.1% owned land up to 5 acres of which lablab was grown on less than 2 acres (71%). Regarding the cultivation of lablab, 71% of the respondents reported having < 10 years’ experience (Table 1).

**TABLE 1:** Description of the respondent’s socio-demographic characteristics.

		Number of Farmers per region						
**Variable**	**Class**	**Arusha**	**Babati**	**Karatu**	**Kondoa**	**Same**	**Total**	**Percentage**

**Gender**	M	6	4	4	5	3	22	71
	F	6	0	1	0	2	9	29

**Age (years)**	<35	2	1	1	1	0	5	16.1
	36–50	4	2	2	3	1	12	38.7
	>51	6	1	2	1	4	14	45.2

**Education level**	Primary	7	2	5	5	4	23	74.2
	Secondary	4	1	0	0	1	6	19.4
	Tertiary	0	1	0	0	0	1	3.2
	Others	1	0	0	0	0	1	3.2

**Occupation**	Farmer	11	2	5	5	5	28	90.3
	Private	0	2	0	0	0	2	6.5
	Others	1	0	0	0	0	1	3.2

**Marital status**	Single	2	0	1	0	1	4	12.9
	Married	9	3	4	5	4	25	80.6
	Widowed	1	1	0	0	0	2	6.5

**Experience (years)**	0–5	8	1	5	0	1	15	48.4
	6–10	1	2	0	1	3	7	22.6
	11–15	0	0	0	2	1	3	9.7
	16–20	0	1	0	0	0	1	3.2
	21–25	0	0	0	2	0	2	6.5
	26–30	2	0	0	0	0	2	6.5
	>31	1	0	0	0	0	1	3.2

**Total land (acres)**	0–2	6	1	2	2	1	12	38.7
	3–5	6	3	2	1	3	15	48.4
	6–10	0	0	1	1	1	3	9.7
	>10	0	0	0	1	0	1	3.2

**Land under lablab (acres)**	0–2	10	2	4	4	2	22	71
	3–5	2	2	0	1	3	8	25.8
	6–10	0	0	1	0	0	1	3.2

**Experience growing lablab (years)**	0–5	8	4	4	0	2	18	60
	6–10	1	0	0	3	3	7	23.3
	11–15	1	0	0	2	0	3	10
	21–25	1	0	0	0	0	1	3.3
	>25	0	0	1	0	0	1	3.3

### Lablab Utilization

**FIGURE 1:**
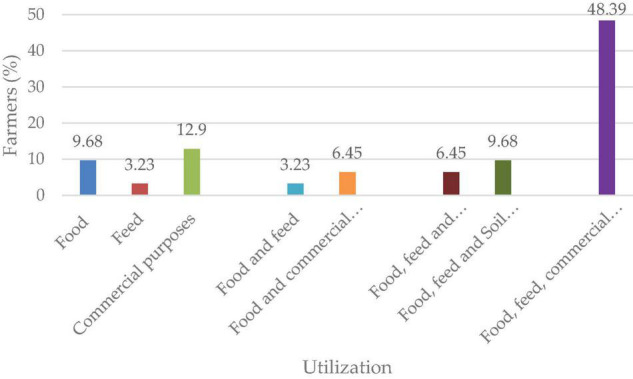
Utilization of lablab by respondents.

It was noted that some of the farmers use lablab for single-purpose, i.e., food (9.68%), animal feed (3.23%), and commercial purposes (12.9%). Farmers from Arusha (6.45%) and Babati (3.23%) mentioned the single-use of lablab. One farmer from Kondoa explained his preference of lablab as animal feed, while 6.48% farmers each from Arusha and Babati grew lablab for commercial purposes. The remaining 64.52% of farmers reported their use for multiple purposes (Figure 1).

**FIGURE 2:**
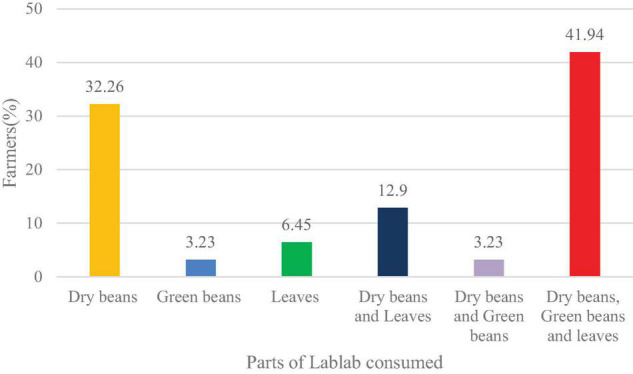
The parts of lablab consumed as food by the respondents.

Concerning the lablab parts consumed, the majority of the respondents use dry beans as food, 3.23% as green beans and 6.45% of them utilize the leaves. The remaining 58.07% respondents consumed a combination of the parts. Of the 32.26% farmers who consume dry beans, 22.58% of them are from Arusha, Karatu (6.45%), and Babati (3.23%). 3.23% of the farmers from Babati described their preference for green beans as food. The remaining 18 farmers use several lablab parts as food (Figure 2).

### Lablab Marketing and Market Information

#### Lablab Marketing Channels and the Peak Selling Season

**FIGURE 3:**
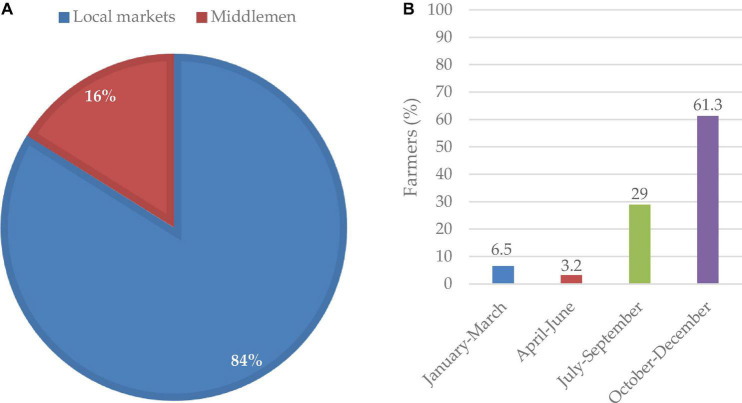
Lablab marketing channels **(A)** and the peak selling period **(B)**.

A majority (84%) of the participants sell their produce in the local market, whereas the remaining 16% sell through the middlemen (Figure 3A). The majority of these farmers reported that the best time to sell their produce was from October to December, followed by July and September (Figure 3B).

#### Source of Market Information for Lablab

**FIGURE 4:**
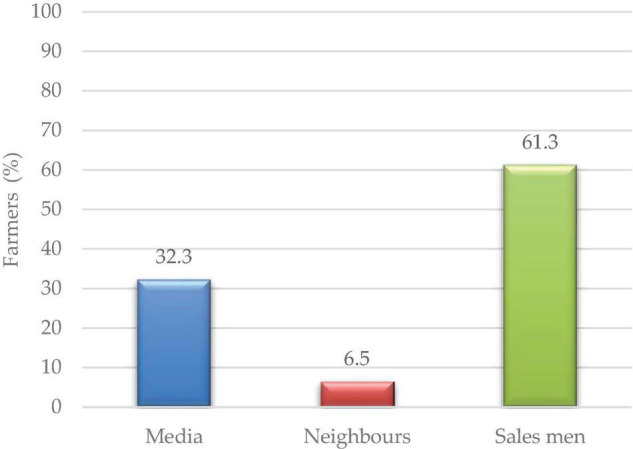
Source of market information.

More than (61%) of the respondents got market information from salesmen, whereas some (32.3%) of the media channels relayed information to farmers, and 6.5% received information from neighbors (Figure 4).

### Source of the Seed

**FIGURE 5:**
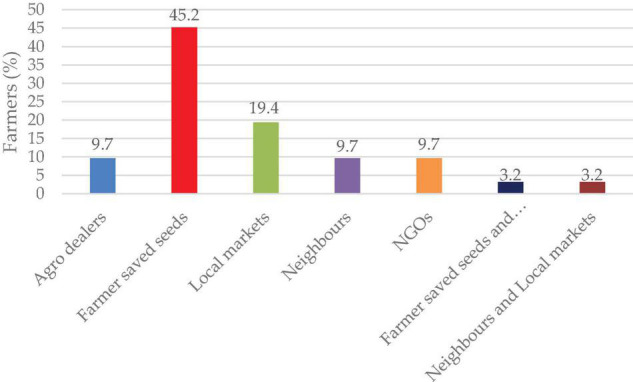
Sources of lablab seeds.

Over 45% of the farmers grow lablab from their own saved seeds from the previous season, followed by purchasing seeds from local markets (19.4%), while agro-dealers, neighbors, and NGOs each constitute 9.7% of the total. Some farmers (3.2%) sourced from both farmer saved and local markets and others (3.2%) from neighbors and local markets (Figure 5).

### Agronomic Practices

#### Source of Information on Lablab Production

**FIGURE 6:**
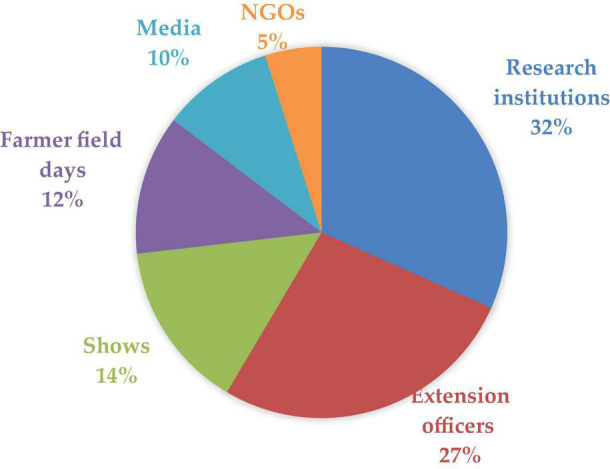
Source of information of Lablab production.

Research institutions were the leading (32%) in disseminating information regarding lablab production. This was followed by the government extension officers (27%), agricultural shows (14%), farmer field days (12%), the media (10%), and NGOs (5%) (Figure 6).

#### Cropping and Harvesting Practices

A majority (58.1%) of the farmers grew lablab as an intercrop, whereas 41.9% grew it as a pure stand. For those that grew lablab as an intercrop, 94.4% reported maize species as the leading intercrop used. Others (5.6%) reported the mix of maize and pigeon peas. Most (83.9%) of the farmers preferred the determinate type of lablab for their production while only 16.1% liked the indeterminate type. Lablab was grown during the long-rain season by a majority of the farmers (93.5%) while 6.5% grew the crop during the short rains. Maturity indices used by most farmers were the pod color (93.5%) while 6.5% used the seed color. During harvesting, a majority of the farmers (90.3%) picked dry pods while 9.7% uprooted the entire plant. Over 80% of the farmers manually threshed their produce while the remaining 19.4% used mechanical methods (Table 2).

**TABLE 2:** Cropping and harvesting practices.

Variable	Interval	Percentage (%)
**Cropping system**	Intercropping	58.1
	Monocropping	41.9

**Intercrop**	Maize	94.4
	Maize, pigeon peas	5.6

**Lablab type**	Determinate	83.9
	Indeterminate	16.1

**Season**	Long Rain	93.5
	Short rain	6.5

**Maturity indices**	Pod color	93.5
	Seed color	6.5

**Harvesting method**	Harvesting dry pods	90.3
	Uprooting entire plant	9.7

**Threshing method**	Manual threshing	80.6
	Mechanical threshing	19.4

### Seed Storage, Sorting, and Color Preference for Food and Commercial Purposes

#### Lablab Seeds Storage Materials

**FIGURE 7:**
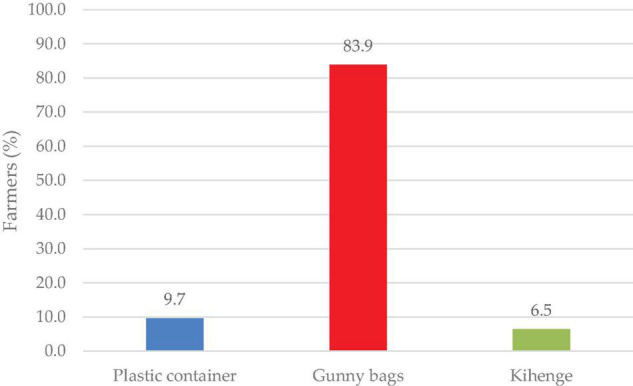
Seed storage materials.

Most (83.9%) of the farmers stored their seeds in gunny bags, followed by plastic containers 9.7% and Kihenge 6.5% (Figure 7).

#### Lablab Seed Storage Time Prior to Planting

**FIGURE 8:**
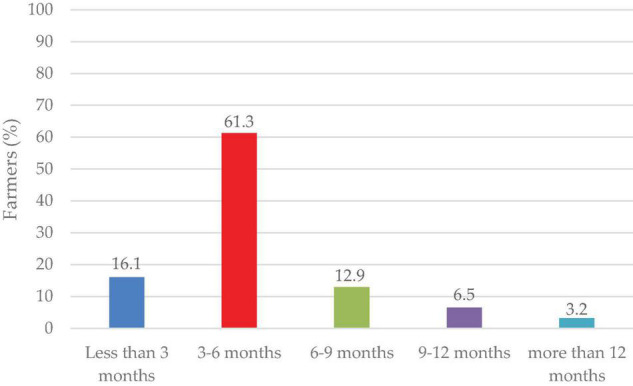
Storage duration (months) of seeds before planting.

The majority (61.3%) of the farmers stored seeds for 3–6 months before planting in the next season, while 16.1% stored their seeds for less than 3 months before planting and only 3.2% stored them for more than 12 months (Figure 8).

#### Seed Sorting

**FIGURE 9:**
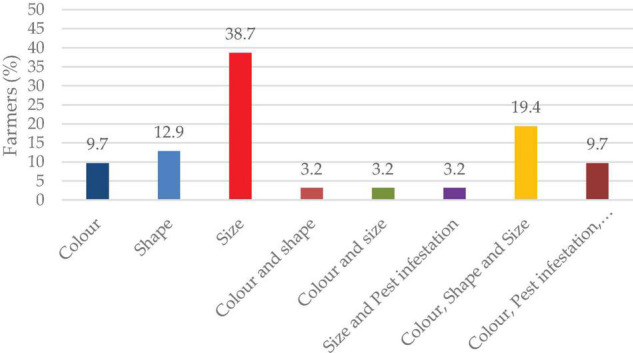
Seed sorting criteria of lablab for planting.

Seed sorting is a critical activity for farmers in determining seeds for the next cropping season. Sorting of seeds based on size is the major criteria used by famers (72.4%). Others attributes such as color and shape (48.3%), diseased seed (10.3%), and pest-infested seed (3.4%) play a critical role. Selection based on the combination of color, shape, and size is followed by 19.4% farmers (Figure 9).

### Farmer’s Food and Market Color Preferences

**FIGURE 10:**
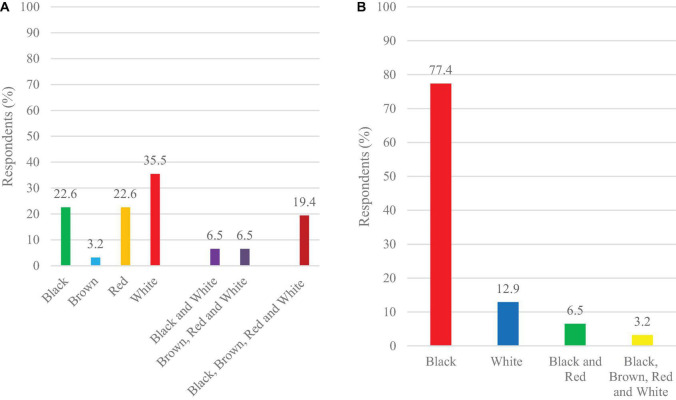
Farmer’s color preferences for **(A)** food and **(B)** commercial purposes.

Preference of white colored seeds for own consumption (35.5%) was reported by farmers, followed by black and red-colored (22.6%), 3.2% preferred brown-colored seeds (Figure 10A). For commercial purposes, the black color (77.4%) is the most preferred, followed by white at 12.9% (Figure 10B).

### Major Constraints in Lablab Production

The major constraints in lablab production mentioned by the farmers across all regions were pests and diseases (83.9%) followed by poor marketability (38.7%). However, poor storage (6.5%) and low yields (19.4%) do not greatly affect lablab production. All the farmers from Same district pinpointed inadequate rainfall as the main challenge in their region. Despite poor storage being least highlighted as a challenge, 6.46% of the farmers, Karatu (3.23%) and Same (3.23%), described poor storage as an impediment to lablab production.

The leading field pests attacking lablab in the field were pod borers (46.9%) and aphids (40.6%). Mites (9.4%) and sucking bugs (3.1%) were also encountered by farmers during lablab production and were highly significant across the study area. Aphids were reported to be found in all the regions under study. Farmers from Babati and Kondoa reported mite infestation while 3.23% of farmers from Kondoa noted sucking bugs as a serious pest. However, in Babati and the Same region, pod borers were not a problem for the farmers. Lablab, despite being a hardy crop, is frequently affected by diseases. According to the farmers, bacterial wilt (38.7%) and late blight (25.8%) are the major diseases during lablab production. Yellowing of leaves was specifically reported by farmers from Same district (19.4%) while powdery mildew was reported by farmers from Arusha, Babati, and Karatu (16.1%). The bruchid (*Callosobruchus* spp.) was the most damaging storage pest (87.1%), causing up to 100% produce loss. The remaining farmers (12.9%) encountered bean weevil attacks during storage. To prevent losses, >83.9% of the farmers applied pesticides before storage, while 12.9% did not apply any chemicals. However, only 3.23% of the farmers from Kondoa used botanicals to control the storage pests. Most of the farmers revealed that they were satisfied with the use of farmer-saved seeds (77.4%) whereas 22.6% expressed dissatisfaction (Table 3).

**TABLE 3:** Major constraints in lablab production.

		Number of Farmers per region						
**Variable**	**Class**	**Arusha**	**Babati**	**Karatu**	**Kondoa**	**Same**	**Total**	**Percentage**

Constraints of lablab production	Pests and diseases	11	3	3	4	5	26	83.9
	Poor marketability	3	3	2	0	4	12	38.7
	Inadequate rainfall	2	1	0	1	5	9	29
	Lack of quality seed	3	2	2	1	1	9	29
	Low yields	1	2	0	1	2	6	19.4
	Expensive agrochemicals	1	2	0	0	3	6	19.4
	Poor storage	0	0	1	0	1	2	6.5

Problematic insects in field	Caterpillar	11	0	2	2	0	15	46.9
	Aphids	1	2	3	2	5	13	40.6
	Mites	0	2	0	1	0	3	9.4
	Sucking bugs	0	0	0	1	0	1	3.1

Serious insects during storage	Bruchids	12	4	4	4	3	27	87.1
	Bean weevils	0	0	1	1	2	4	12.9

Lablab diseases	Bacterial wilt	5	2	0	5	0	12	38.7
	Late blight	5	0	3	0	0	8	25.8
	Yellowing	0	0	1	0	5	6	19.4
	Powdery mildew	2	1	2	0	0	5	16.1

Pest control	Pesticides	12	4	4	3	3	26	83.9
	No application	0	0	1	1	2	4	12.9
	Botanicals	0	0	0	1	0	1	3.2

Seed Treatment	Yes	9	3	3	1	5	21	70
	No	2	1	2	4	0	9	30

Satisfaction with farmer saved seeds	Yes	9	3	2	5	5	24	77.4
	No	3	1	3	0	0	7	22.6

### Farmer Preference for Lablab Improvement

**FIGURE 11:**
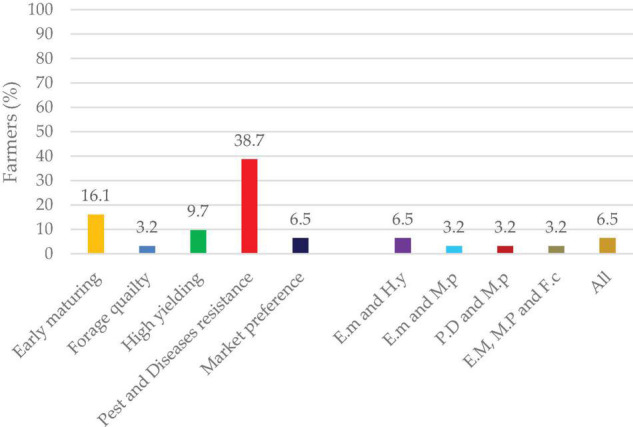
Farmers’ preferred traits for variety development.

The farmers enumerated six traits of preference to be of importance in their lablab production. A majority of the farmers reported improvement of pests and disease resistance as the most important trait for consideration (38.7%). This was followed by early maturity varieties (42.9%), while the least was forage quality (7.1%). A combination of all these traits to be improved was also reiterated by farmers (6.5%) (Figure 11).

### Farmer Field Selection

**FIGURE 12:**
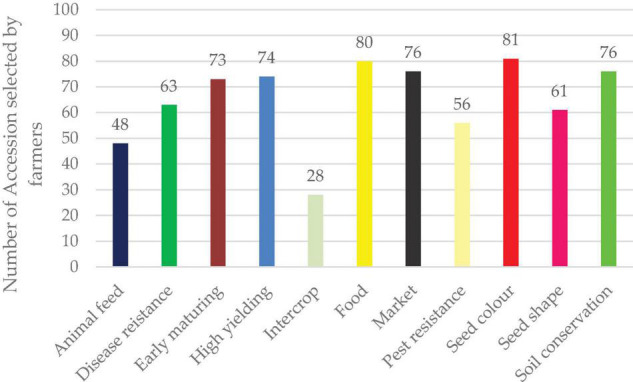
Preferred trait selection by farmers.

Accessions evaluation for 11 traits was done by farmers in the experimental field. The leading traits with many accession selections were seed color (81) and food (80) while the traits with the least selections were intercrop (28) selections (Figure 12).

### Best Five Preferred Selections Per Category

**FIGURE 13:**
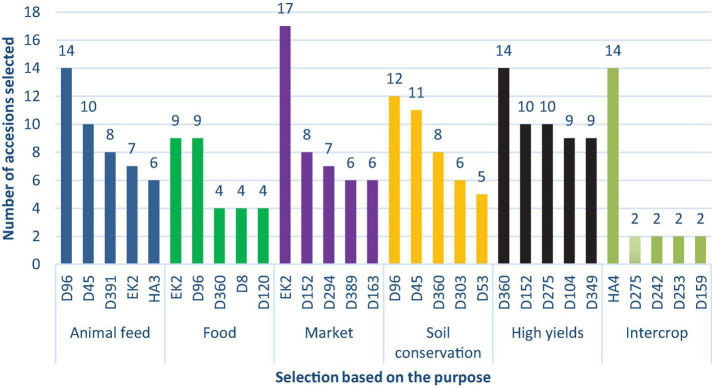
Selection of five accessions based on their uses.

**FIGURE 14:**
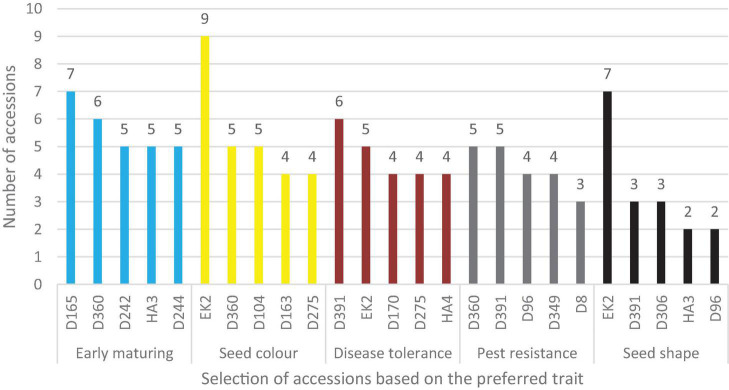
Selection of five accessions based on preferred traits.

The selected farmers were invited to rate the accessions that had been planted in the field as per the traits in Figure 12. The best five varieties with the highest frequency based on uses (Figure 13) and based on preferred traits (Figure 14) were recorded. For animal feed, 14 farmers selected D96 followed by 10 farmers who selected D45, while for food consumption EK2 and D96 were selected by 9 farmers each. In terms of commercial purposes, EK2 was selected by 17 farmers, whereas the other four accessions were selected by at least six farmers. Two accessions (D96 and D45) were selected by more than 11 farmers for soil conservation purposes. One accession, D360, was selected by 14 farmers for high yielding potential, whereas 14 farmers also selected HA4 for its use as an intercrop.

In terms of preferred traits, accession D165 was selected by at least 7 farmers as early maturing, followed by D360, D252, HA4, and D251, respectively. Three accessions, D360, D152, and D287 were selected by more than 10 farmers as high yielding. Six farmers were able to identify D391 as a disease tolerant species, followed by EK2, D170, D287, and HA4. Similarly, D360 and D391 were identified by at least 5 farmers as being pest resistant. In regards to seed shape, EK2 was preferred by majority of farmers (Figure 14).

## Discussion

### Socio-Demographic Characteristics

This study was carried out to determine the farmers’ knowledge, practices, production constraints, and trait preference of lablab in selected regions of Tanzania. The information from this study forms a basis for lablab breeding and improvement tailored toward farmer’s needs. Lablab production in the selected areas was male-dominated (Table 1). This is because males are regarded as the main decision-makers in matters related to family, land and production. Land ownership in Tanzania is guided by customary laws and traditions and is mostly under control of patriarchal norms. Female ownership is majorly determined by their husbands’ willingness to give land for production (Moyo, 2017; Lusasi and Mwaseba, 2020). Lack of access to resources such as land and power of decision-making has reduced women’s influence in crop production (Ishengoma, 2004). This study, however, contradicts a similar study by Chawe et al. (2019) on lablab production in the Northern part of Tanzania, who reported a greater percentage of females in lablab production as compared to males. The findings from our study could be related to the purpose as a commercial crop and thus male are more involved in the production.

Most of the farmers involved in lablab production were >36 years old and this could be associated with rural-urban migration by the younger generation perceiving agriculture and related activities as poor-paying (Wineman et al., 2020). This study is in agreement with several others (Chawe et al., 2019; Lindsjö et al., 2020) who reported the involvement of older generations in lablab production. Over 74% of the farmers had basic level education. Abed et al. (2020) revealed similar results in farmers’ literacy levels. Farmer knowledge and education play a key role in enhancing production. This indicates the ability to interpret written material, posters, pamphlets, and even listen and contextualize audio material distributed by government officials, research institutions and NGOs like East Africa Impact Centre (ECHO) and Feed the future program (FANAKA) (Abed et al., 2020). Increased literacy levels are directly correlated to increased adoption rates of new technology and even varieties in crop production.

Similarly, the contribution of labor by both husband and wife would increase productivity as compared to single individuals (Kimaro et al., 2013; Ngongi and Urassa, 2014). Farmer experience in crop production is a critical factor that determines and impacts positively on productivity. Experience influences the ability to access information and expertise that can enhance crop productivity (Makawia, 2018). A majority of the farmers interviewed had <10 years’ experience, indicating little knowledge and information related to lablab. As a neglected and underutilized crop, information on lablab production is limited (Suvi et al., 2021), which may be due to a lack of experience in its production. Land ownership dictates the farmers’ ability to make decisions, access to financial resources and incentives, and the ability to efficiently and sustainably use land and adopt new technologies (Abed et al., 2020). The present study established that a majority of the farmers possessed <5 acres of land, with most of them growing lablab in <2 acres. These small-holder farmers are disadvantaged in accessing resources since financing institutions regard them as risky ventures for credit facilities. These results are consistent with the assessment of rice farmers in Tanzania (Suvi et al., 2021). Similarly, participants with larger land holdings are more likely to obtain higher yields, which are linked to economic benefits, when compared to small-holder farmers (Kulyakwave et al., 2019). The small acreage allocated for lablab production explicitly explains the neglect of its production as it is still considered of less value as compared to the other crops.

### Crop Utilization

This study reports that the majority of the farmers utilize lablab for different purposes. From the study, some farmers use lablab for a single purpose as human food, animal feed and commercial purposes. Most of the interviewed participants utilize lablab as food in their households. Ewansiha et al. (2016a) reported that farmers were adopting lablab as food due to its nutritional value. Similar findings have been reported (Kankwatsa, 2018). Its use as animal feed where it is grazed directly by livestock or can be harvested to make hay and make silage has been reported (Manyawu et al., 2016; Bhardwaj and Hamama, 2019; Wangila et al., 2021). Lablab is regarded as an economically valuable crop due to the prices it fetches in the market. Its importance for commercial purposes was emphasized by farmers (Figure 1). The high lablab seed prices in Kenya have contributed to the adoption of lablab production by Tanzanian farmers in the selected regions leading to a gradual shift by farmers toward lablab cultivation. Increased awareness and production of Lablab will result in market creation and subsequent increased economic benefits due to its sale. Soil conservation is critical to the farmers to allow increased productivity with farmers adopting lablab crop in their production systems (Massawe et al., 2016; Lasway et al., 2020).

The parts of lablab consumed vary from the dry beans, leaves, green beans, and fresh pods (Figure 2). The present study revealed that most of the farmers consumed dry beans, which are usually considered highly nutritious as compared to those of other legumes. Davari et al. (2018), Morrison (2019), Purwanti et al. (2019), and Kilonzi (2020) reported that the leaves of lablab are edible with high nutritional factors. Leaves were also reported by the farmers in the present study as their preference for food, especially during the dry season when other vegetables are not growing in the gardens (Shetto and Owenya, 2007; Voss, 2013; Banjarnahor, 2014). Our study also found out that the fresh beans were being utilized by the farmers as food. Fresh beans contain potassium, sodium, calcium, zinc, magnesium, manganese and copper that are essential in the human body (Guretzki and Papenbrock, 2014; Morrison, 2019). None of the farmers consumed the fresh pods of lablab as food. In Asian countries, fresh pods are the main parts of consumption in lablab (Rai et al., 2014; Moonmoon and Ahmod, 2020).

### Marketing of Lablab Produce

Lablab is regarded as a minor crop and its market system is not still well described in Tanzania. Most of the respondents sold their produce to local markets, attributing to the poor marketing channels and knowledge on the pricing, and commodity availability. Forsythe (2019) emphasizes the need to revitalize lablab production with a focus on improving marketability and pricing to make it an attractive venture for farmers. Farmer assessment by Chawe et al. (2019) also revealed similar challenges of poor market demand as elaborated by the farmers. The middlemen involved set low prices for the lablab produce resulting in very low-profit margins for the farmers, yet the same product has higher profit margins in international markets. The low-profit margins drive the farmers to shift to higher-valued crops with increased profit margins (Forsythe, 2019). According to Mabhaudhi et al. (2019) fragmented and disorganized markets and variability of pricing hamper adoption and production of neglected and underutilized crops. Market demand for lablab is high during October-December and this is the period when farmers can maximize their profits (Kinyua et al., 2008).

Salesmen/traders were identified as the main source of information on market demands and availability (Figure 4). Limited access to market information has been singled out as the cardinal bottleneck to lablab production (Mkuna and Temu, 2016; Forsythe, 2019). Deliberate efforts should be made to increase the dissemination of lablab information to farmers to enable them to make informed decisions regarding their produce. The middlemen, who are also known as “brokers” purchase the product at the farmers’ gates at low prices and sell at exorbitant prices achieving higher profit margins at the expense of the farmers. New and expanded market opportunities will attract and increase the production of lablab crops in the country.

### Seed Source

Informal seed systems contribute to > 90% of the legume crops grown by small-holder farmers in Sub-Saharan Africa (Akpo et al., 2020). Farmers prefer their seeds (Figure 5) due to low cost, readily available during planting season, desired characteristics, and known quality since they are the custodians. This also involves farmer to farmer seed transfers usually based on one’s demand and existing social relationship with each other (ASARECA/KIT, 2014). The participants highlighted farmer-saved seeds as the common seed source for their production. In Kenya, research institutions such as Kenya Agricultural Livestock Research Organization (KALRO) Katumani Dryland Research Station have released lablab varieties such as KAT-DL1, KAT-DL2, and KAT-DL3 (Kilonzi, 2020), Eldoret KT-Maridadi, Eldoret KT Black 1, Eldoret KT Black 2, and Eldoret Cream at the University of Eldoret that describe the formal seed system. These seeds are sold through registered agro-dealers, increasing farmer accessibility. The other seed sources, such as neighbors, NGOs, and local markets, were outlined by farmers as the alternative seed sources. NGOs such as ECHO and Rekolto, Tanzania have been carrying out intensive awareness of lablab together with its multipurpose use as a Conservation Agriculture (CA) crop, leading to cultivation by farmers Mariki and Miller (2017). The development and release of certified seed quality enhances production, which directly relates to increased profits to the farmers.

### Information on Production Practices of Lablab

Agricultural research information needs to be diffused to the end-user at an appropriate time, proper channels, and in simplified and farmer-customed language for ease of adoption and eventual execution at the farm level (Figure 6). Tanzania has established 17 agricultural research institutes that have been mandated to undertake research all over the country.^2^ These research institutions are involved in the transfer of research information, adoption of improved technologies, varieties, and modern practices that can easily be adopted by farmers (Mubofu and Elia, 2017). University institutions such as the Nelson Mandela African Institution of Science and Technology (NM-AIST), the University of Dar es Salaam, Iringa University, Sokoine University of Agriculture (SUA), and the University of Dodoma generate significant findings which are farmer centered and the diffusion of this information to the end-user means enhanced crop improvement. Agricultural shows and exhibitions such as Sabasaba and Nanenane have been fronted by the government to disseminate agricultural information, technologies, and innovations to farmers. Research institutions, learning institutions, seed companies, and other agriculture-related sectors transfer and share research findings with farmers enhancing knowledge diffusion to farmers at the local level (Wetengere, 2016).

Agricultural knowledge and information are transferred through various media forms which include mobile phones, audio, visual, and print media. Tanzania boasts of the existence of various mobile operators which enhance the knowledge transfer to farmers. Tigo Kilimo (Tigo), Zantel Kilimo (Zantel), and Kilimo Club (Vodacom) (Global System for Mobile Communications Association, 2015) highlight the use of mobile phones in the dissemination of information to farmers (Ndimbwa et al., 2020). Other studies have also reported radio to be the primary source of information followed by television that enables the spread of information to small-holder farmers in rural communities (Isaya et al., 2018; Mtega, 2018; Mubofu and Malekani, 2020). Improved access to mass media platforms could be the springboard to enhancing farmer access to information and evidence-based interventions.

### Cropping System and Harvesting of Lablab

The present study revealed that lablab was intercropped with maize and pigeon peas (Table 2). Intercropping lablab with other crops improves yield (Nord et al., 2020; Aluku et al., 2021), lowers insect pest abundance (Forsythe, 2019), improves soil quality (Maass et al., 2010), and maximizes the use of land resources (Massawe et al., 2016). Cultivation of lablab as a sole crop occupies land space that can also be used for other crops. The indeterminate growth cycle of lablab that can range from 3 to 7 months prevents farmers from adopting its full cultivation (Gamachu, 2018; Ayele, 2020; Rapholo et al., 2020). Determinate crop varieties are preferred by farmers, especially with the small land size (Chawe et al., 2019) and are suitable for intercropping. Lablab production was mainly done during the long rain season that occurs between March and June. The production of lablab during long-rain seasons has also been reported (Nord et al., 2020; Aluku et al., 2021), with a tenfold increment in yield performance compared to cowpeas, pigeon peas and common beans. Other farmers are, however, involved in lablab production during the short-rain season since lablab a drought resistant crop is. Commonly, maturity indicators in lablab depend on the change of pod color from green to brown (Athmaselvi et al., 2020). This observatory indicator has been used by farmers together with the drying-up of the crop vines to determine the timing and maturity of the crop. Proper identification of maturity in lablab impacts the total yield harvested. Most farmers harvest only the pods which are then manually threshed (Athmaselvi et al., 2020). Manual threshing in these rural communities is preferred due to the availability of economical manpower.

### Seed Storage, Sorting, and Color Preference for Food and Market

Most of the farmers stored their seeds in gunny bags for the next season (Figure 7). Poor storage facilities were reported as a constraint by farmers involved in lablab production (Chawe et al., 2019). Proper storage techniques and materials affect the seed viability, vigor, and development when sown in the field. In addition to the gunny bags, traditional grain storage was done on “kihenge.” Kihenge refers to traditional storage constructed from bamboo, reeds and mud. This method is considered low-cost, affordable and does not require technical skills to construct (Luoga, 2019). Most farmers store their seeds for 3–6 months before planting in the field since the seed germination, viability and vigor are at their optimum at this period (Figure 8). Farmers sorted their seeds for the next season based on sizes (Figure 9). Large-sized seeds contain more food reserves as compared to small-sized which is directly correlated to early seed establishment and eventual development (Singh et al., 2017). Farmers’ selection was based on indigenous skills passed from one generation to another on the decision of selecting a particular seed for the next planting season. Seed selection based on color was mostly for the market and food consumption (Figures 10A,B). Most of the farmers preferred white-colored varieties for consumption while, black colored for market purposes (Ewansiha et al., 2007; Morrison, 2019). Studies have revealed that consumers use visual appearance when making choices for consumption (Maina, 2018). Conversely to our results, Morrison (2019) reported that Karamoja Red, a red colored seed, was the most preferred accession for consumption by farmers. Despite its origin being Uganda, this variety has been cultivated by most farmers in Tanzania and its awareness could have contributed to its preference by farmers selected for the present study.

### Major Constraints on Lablab Production

The farmers identified the constraints that impede lablab production as insect pests and diseases, inadequate rainfall, poor marketability, poor seed quality, expensive agrochemicals, low yields, and poor storage (Table 3). All farmers from the five regions pinpointed insect pests and diseases as key factors that hinder their involvement in the production of lablab. However, the farmers from Same further emphasized the low rainfall as the main hindrance in their district. Same is a semi-arid district in the northern part of Tanzania and has been reported to receive inadequate annual precipitation of 890 mm to support crop production (Tumbo et al., 2012). Poor marketability contributes to its neglect as a crop by farmers (Chawe et al., 2019).

The major insect pests that infest lablab in the field include pod borers, aphids, sucking bugs, leaf miners, flower thrips, mites, pod borers, and sucking pests (Kamotho, 2015; Ewansiha et al., 2016b; Nahashon et al., 2016; Sambathkumar et al., 2017; Forsythe, 2019). The farmers reported pod borers, aphids, mites, and sucking bugs as the most prevalent pests in their production zones. Pod borers attack the crop at the reproductive stage, resulting in poor pod formation and setting as well as an increased cost of production due to the requirement to do spraying (Shinde et al., 2017). Several species of aphids have been reported in lablab and are usually associated with destructive damage and acting as a vector for most viral diseases (Chopkar et al., 2020). Their infestation varies greatly depending on geographical location and climatic conditions (Reddy et al., 2017; Bharathi et al., 2020). High population levels occur during the flowering to pod formation stage (Golvankar et al., 2019). Breeding of resistant varieties for aphid and pod borers can help catapult the development, adoption and utilization of lablab since they are regarded as the leading pest.

Farmers from Babati and Kondoa reported attacks by mites. This was also documented by Sarwar (2019) and Khan et al. (2020). Despite some mite species being harmful, some species have been described to act as a biological control for other insect pests (Sarwar, 2019). Proper identification and characterization of mite species can provide an effective integrated pest management alternative. Sucking bugs have been reported to cause considerable damage to lablab production (Khan et al., 2020). Proper insect pest management of this pest can increase lablab production. During storage, farmers mentioned the bruchid pests as the most devastating and could even lead to total crop loss. Worldwide, bruchid attacks have been reported to attack crops both in the field and during storage of pulses (Prasad et al., 2013). Proper storage methods are required to prevent loss of produce. The use of triple-layer hermetic storage is inexpensive, safe, and economical compared to the use of insecticides that are costly, unsafe, and harmful to the environment (Vanitha et al., 2018). Development of pest resistant varieties has been pinpointed as the alternative management strategy.

Bacterial wilt, late blight, yellowing of leaves, and powdery mildew were highlighted by the farmers as the most common diseases. Bacterial wilt is a major problem in lablab affecting young plants and seedlings, resulting in wilting, mortality and severe crop loss (Osdaghi et al., 2020). Being a seed-borne disease, it is easily disseminated to various places and its management is crucial for lablab production. Planting of resistant varieties should thus be a priority to efficiently manage the disease (Harveson et al., 2015). Other diseases of lablab are the yellow mosaic virus (Suruthi et al., 2018), and *Colletotrichum* spp (anthracnose) (Ewansiha et al., 2016b). The use of synthetic chemicals increases the cost of production and thus lowers the profit margin for farmers. While the use of botanicals can act as an alternative only one farmer reported use of botanicals in pest control. A few studies have been done on botanical use in the control of bruchids (Rugumamu, 2014; Sanon et al., 2018; Ekoja and Ogah, 2020; Govindan et al., 2020), which demonstrates the increasing research toward solving societal challenges using affordable and cheap materials at the community level.

### Farmer Preferred Traits for Lablab Improvement

Farmer-focused breeding entails incorporating the traits of concern into the research programs leading to the development of varieties that farmers can easily adopt. In the present study, farmers prioritized the development of insect-pest and disease resistance, early maturing varieties, high yielding, market preference, good cooking ability, and improved palatability as forage crop, respectively (Figure 11). Breeding of varieties resistant to pod borer and bruchids can pave the way for increased production of lablab with minimal cost of production, which in turn relates to increased economic benefits for farmers (Prasad et al., 2013; Boit et al., 2018). The development of early maturing varieties can also allow the plant to complete its life cycle early and avoid instances of drought spells in the field. Late and indeterminate varieties are considered unfavorable to farmers due to their increased cost of production in terms of weeding and chemical applications to control insect-pests which affects the establishment of the main crop in the fields. Yield attributes play a big role in selecting the varieties to be grown in the field. High yields directly relate to more income for the farmers and hence, improved standards of living. Market preference (Morrison, 2019), good cook ability (Kankwatsa, 2018; Morrison, 2019), and forage quality (Ewansiha et al., 2016a) are other important attributes that require intensive research and be tailored to meet farmers’ selection criteria.

### Farmer’s Field Selection Study

Farmers selected accessions according to the 11 parameters pre-defined for them. From the planted accessions in the field, the trait with the most selections was seed color and food. Seed color is a critical attribute when selecting a lablab variety. Farmers prefer cultivation of black colored accessions for market purposes whereas white/cream color is majorly considered for food consumption. Dark seeded lablab varieties are associated with bitter taste as compared to their light-colored counterparts (Shivachi et al., 2012) thus influencing the farmers’ choice of growing a particular accession. Despite lablab being neglected, farmers selected 80 accessions they considered suitable for food consumption. As earlier explained, lablab is an important nutritional security crop which if adopted, can help solve the food security crisis faced by many countries in Sub-Saharan Africa (Minde et al., 2020). Cultivation of lablab as a soil conservation strategy has been done in various regions including Tanzania. In fields in which lablab has been grown, there was increased water infiltration, minimal soil erosion and reduced evaporation (Owenya et al., 2011). Some farmers grow lablab for economic reasons, particularly in Kenya and Rwanda, where demand is increasing (Shumbusha et al., 2020). The least number of accessions selected was for intercrop purposes. Most existing accessions exhibit spreading growth habits and are unsuitable for use as intercrops (Maass et al., 2010). Breeding of varieties with erect growth habits suitable for use as an intercrop is important, especially for smallholder farmers involved on mixed farming in limited land resources.

The 5 most preferred traits with the highest frequency selected by farmers were tabulated as in Figure 13. Accession D96 and D45 were the leading accessions selected by farmers as animal feed due to their dense foliage, which relates to high fresh and dry matter weight, which is critical in determining the choice of forage crop (Ewansiha et al., 2016a). EK2 and D96 were considered by most farmers for food consumption. Despite being black and brown seeded, their selection was based on pod characteristics and grain type. EK2 and D152 were considered for market purposes, both being dark-colored. As earlier highlighted, color is a factor in selection of a given variety for market or food consumption. D96 and D45 were identified as potential accessions for soil conservation due to their high biomass that covers the surface, thus curbing soil erosion. Dense ground cover plays an important role in the conservation of land resources. Breeding of lablab varieties with dense foliage is vital, especially with current changing climatic patterns. High yielding is critical when selecting a particular variety for breeding. In the present study, D360, D152, and D275 were identified by farmers as potential high-yielding breeding materials. An increase in yields is directly associated with economic returns, which in turn relate to improved livelihoods. One accession (HA4) was identified by farmers as potential breeding material for intercrop use. Accession HA4 has an erect growth habits hence making it suitable for intercropping with other crops such as maize or sorghum. This variety was bred for its determinate growth habit and photoperiod sensitiveness (Keerthi et al., 2014). Breeding of more accessions to be used for intercrop use is required.

Trait preference in development of a given variety is key to its adoption, utilization and production. Farmers identified D165 and D360 as early maturing accessions and further breeding should be done on them. These varieties have a short life cycle, the ability to efficiently maximize water and fertilizer resources and allow economical land use (Grotelüschen, 2014). In terms of seed color and seed shape, EK2 was selected by the majority of the farmers. The seeds of this variety are round and black in color. This attribute is considered suitable for market value. Seed shape and uniformity are critical attributes for machine harvesting, especially in large tracts of land. Development of pest and disease resistant varieties is a core aspect for lablab farmers. D391 and EK2 were identified as potential disease-tolerant varieties, whereas D360 and D391 exhibited pest-resisting ability. Resistant varieties reduce the use of chemicals thus reducing the cost of production, environmental sustainability, and food and human safety (Harveson et al., 2015). Breeding of resistant varieties is thus paramount in lablab breeding program. Mother and baby trials on accessions selected by farmers have been reported to foster adoption and dissemination of a crop (Najeeb et al., 2018). Focusing breeding programs and objectives on these farmer-selected accessions will lead to the release and selection of genotypes with farmer preferred traits.

## Conclusion

Lablab is considered a climate-smart crop that can contribute to food and nutritional security and help sub-Saharan Africa combat the perennial challenges of malnutrition and climate change effects. Despite this, the production of lablab is faced with a myriad of challenges, such as pest and disease attacks, inadequate rainfall, poor marketability, and poor cooking value. Being an underutilized crop, lablab lacks a formal seed system, with farmers depending on farmer-saved seeds. Poor marketing channels result in low prices that, in turn translate into reduced profits to the farmers. Farmer adoption of a given variety largely depends on the presence of the desired traits in that variety. Farmer’s preferred traits such as developing pest and disease-resistant varieties and high-yielding varieties should be incorporated into the breeding programs of lablab. Promising accessions such as EK2, D360, HA4, and D96 could be considered for further multi-locational trials and breeding. This study generates vital information for researchers, breeders, and policymakers toward enhancing production, consumption, and utilization of lablab.

## Data Availability Statement

The original contributions presented in the study are included in the article/supplementary material, further inquiries can be directed to the corresponding author.

## Author Contributions

PV: conceptualization and designing the experiments. FL: experimentation, data collection, data analysis, and manuscript writing. PN: supervision, reviewing, and editing the manuscript. PV: final internal review and revising the final draft of the manuscript. All authors contributed to the article and approved the submitted version.

## Conflict of Interest

The authors declare that the research was conducted in the absence of any commercial or financial relationships that could be construed as a potential conflict of interest.

## Publisher’s Note

All claims expressed in this article are solely those of the authors and do not necessarily represent those of their affiliated organizations, or those of the publisher, the editors and the reviewers. Any product that may be evaluated in this article, or claim that may be made by its manufacturer, is not guaranteed or endorsed by the publisher.

## Footnotes

1
http://www.iasri.res.in/design/augmented%20Designs/home.htm

2
www.tari.go.tz
